# MicroRNAs expressed by human cytomegalovirus

**DOI:** 10.1186/s12985-020-1296-4

**Published:** 2020-03-12

**Authors:** Lichen Zhang, Jiaqi Yu, Zhijun Liu

**Affiliations:** 1 Clinical School, Weifang Medical University, Weifang, 261053 China; 2 Department of Medical Microbiology, Weifang Medical University, Weifang, 261053 China

**Keywords:** Human Cytomegalovirus (HCMV), miRNAs, Latent infection

## Abstract

**Background:**

MicroRNAs (miRNAs) are small non-coding RNAs about 22 nucleotides in length, which play an important role in gene regulation of both eukaryotes and viruses. They can promote RNA cleavage and repress translation via base-pairing with complementary sequences within mRNA molecules.

**Main body:**

Human cytomegalovirus (HCMV) encodes a large number of miRNAs that regulate transcriptions of both host cells and themselves to favor viral infection and inhibit the host’s immune response. To date, ~ 26 mature HCMV miRNAs have been identified. Nevertheless, their roles in viral infection are ambiguous, and the mechanisms have not been fully revealed. Therefore, we discuss the methods used in HCMV miRNA research and summarize the important roles of HCMV miRNAs and their potential mechanisms in infection.

**Conclusions:**

To study the miRNAs encoded by viruses and their roles in viral replication, expression, and infection will not only contribute to the planning of effective antiviral therapies, but also provide new molecular targets for the development of antiviral drugs.

## Background

Herpesviruses belong to a ubiquitous family of enveloped dsDNA viruses and are subdivided into three subfamilies (α, β and γ), based on their sequence homology. Human cytomegalovirus (HCMV) belongs to the β-herpesvirus subfamily. HCMV inhibits virus replication and release by suppressing its own gene expression, thereby achieving latent infection. Meanwhile, the virus is ready to reactivate upon appropriate stimulation. The ability of HCMV to establish a latent infection, undergo periodic reactivation, and evade the host immune response determines whether it successfully infects the host. It has been reported that in developed countries ~ 60% of adults have antibodies of the IgG class against HCMV, while this number in developing countries is nearly 100% [1]. Fetuses and immunocompromised patients such as those with acquired immunodeficiency syndrome, after organ transplantation, or with cancer, may suffer severe disease after infection with HCMV, including mononucleosis-like syndrome, interstitial pneumonia, gastroenteritis, and retinitis [2, 3].

MicroRNAs (miRNAs) are ~ 22 nucleotides long non-coding RNAs encoded by eukaryotes and viruses and can regulate gene expression. Since Lee and colleagues identified the first miRNA in *Caenorhabditis elegans* in 1993 [4], at least 48,000 mature miRNAs have been identified [5]. In general, miRNA genes are clustered and transcribed from polycistronic genes [6]. They are regulated by their own upstream regulatory sequences [7]. Mature miRNAs participate in the formation of an RISC (RNA-induced silencing complex). The RISC-loaded miRNA binds a sequence within the target mRNAs. When the seed sequence of miRNA is completely complementary to its binding sites, it causes mRNA degradation. In contrast, translation is inhibited if a miRNA has an imperfect match to the target mRNA. Although mature miRNA sequences derived from each arm of the hairpin precursor may have their own biological functions, in most cases, only one strand is incorporated into the RISC, and the dominant mature sequence depends on the developmental stage or tissue [8].

**Fig. 1:**
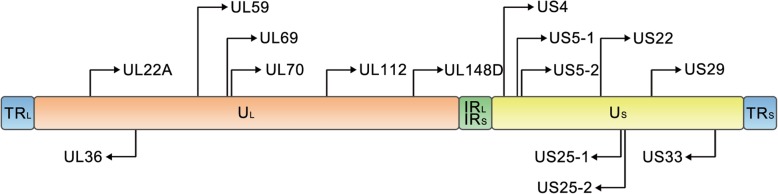
HCMV genome and the genomic distribution of HCMV miRNAs. The HCMV genome is divided into unique long (UL) and unique short (US) regions, and these two regions are flanked by terminal and internal inverted repeats (TRL, IRL, TRS, and IRS). The locations of HCMV pre-miRNAs are shown on the genome. Sense and antisense miRNA precursors are distinguished by the orientation of the arrows

**Fig. 2:**
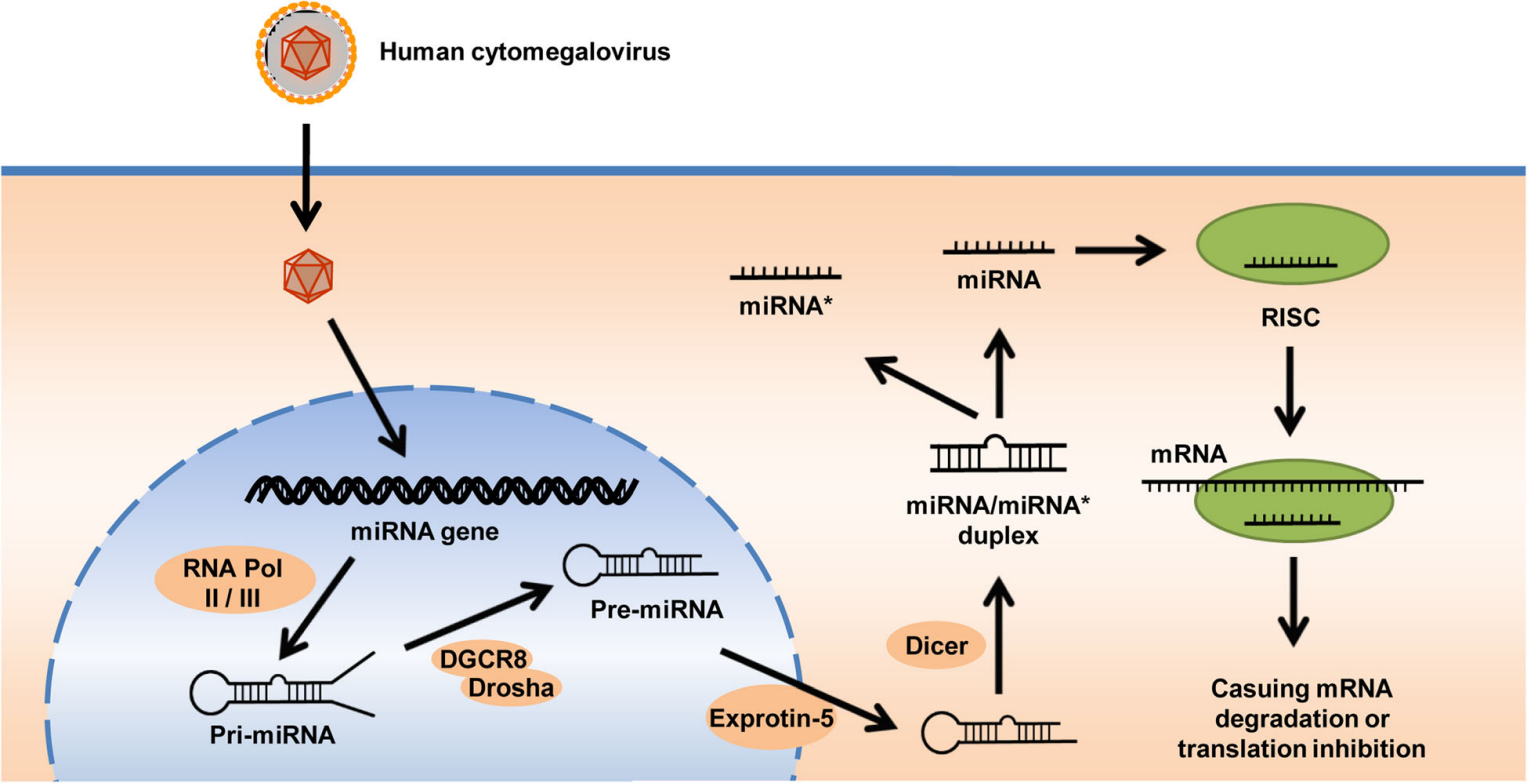
Biogenesis of miRNA. First, the HCMV miRNA gene is transcribed to generate RNA transcript called primary (pri)-miRNA by RNA polymerase (Pol) II or III. Second, pri-miRNA is processed to precursor (pre)-miRNA by DGCR8 and RNase III Drosha. Third, pre-miRNA is exported to the cytoplasm by Exportin-5, then cropped to the miRNA/miRNA* duplex by the RNase III enzyme Dicer. Finally, one strand of the duplex, miRNA*, is unwound while the other strand is further processed, giving rise to a mature RISC (RNA-induced silencing complex).to bind mRNA, causing mRNA degradation or inhibiting translation

Viruses encode miRNAs that regulate the gene expression of host cells and viruses in order to generate a more favorable cellular environment or to inhibit the host’s immune response [9, 10]. The first set of viral miRNAs were identified by Pfeffer et al. in 2004 in Epstein-Barr virus [11]. To date, ~ 500 viral miRNAs have been reported (according to miRBase 22, http://www.mirbase.org). The majority of these miRNAs are encoded and expressed by herpesviruses [12], such as HCMV (Fig. 1), Epstein-Barr virus, and Herpes simplex virus. A notable characteristic of herpes viruses is that they can use viral proteins and viral miRNAs to establish a lifelong latent infection in their host without producing overt disease [13]. These miRNAs cooperate with viral proteins to regulate the expression of viral and/or host genes that are involved in the immune evasion, survival, and proliferation of infected cells, as well as, critically, the latency and reactivation of the virus. So far, ~ 26 mature HCMV miRNAs have been reported, along with their potential targets (Table 1). Interestingly, in contrast to other herpes viruses, the miRNA genes of HCMV are scattered throughout the viral genome (Fig. 2), implying that the expression and function of each isolated HCMV miRNA may be regulated by its own regulatory sequence. In this review, we summarize the important roles of HCMV miRNAs and their potential mechanisms in infection, as well as discussing the research methods used to investigate HCMV miRNAs.

**Table 1:** Currently known HCMV miRNAs and/or potential miRNAs targets and their functions

Pre-miRNA names	Mature miRNA names	Sequences	Targets	Main Function
mir-UL112	miR-UL112-3p	aagugacggugagauccaggcu	UL114 [14]	escape immune elimination and induce viral latency
			BCLAF1 [15]	
			MICB [16]	
			MICA [17]	
			UL112/113 [18]	
			UL120/121 [18]	
			IE72 [19]	
			IRF1 [20]	
			VAMP3 [21]	
			RAB5C [21]	
			RAB11A [21]	
			SNAP23 [21]	
			CDC42 [21]	
			ATG5 [22]	
			IKKα/β [23]	
			IL32 [24]	
			TLR2 [25]	
	miR-UL112-5p	ccuccggaucacaugguuacuca	ERAP1 [26]	escape immune response
			CASP3 [22]	
mir-UL148D	miR-UL148D	ucguccuccccuucuucaccg	RANTES [27]	escape immune response and regulate apoptosis of host cells
			IEX-1 [28]	
			ACVR1B [29]	
			ERN1 [30]	
			PHAP1 [30]	
mir-UL22A	miR-UL22A-3p	ucaccagaaugcuaguuuguag	CASP7 [22]	participate in cell differentiation and immunity
			SMAD3 [31]	
	miR-UL22A-5p	uaacuagccuucccgugaga	BMPR2 [32]	
			CASP3 [22]	
			SMAD3 [31]	
mir-UL36	miR-UL36-3p	uuuccagguguuuucaacgugc	CDK6 [22]	N/A
			FAS [22]	
	miR-UL36-5p	ucguugaagacaccuggaaaga	UL138 [33]	contribute to HCMV replication
			SLC25A6 (ANT3) [34]	
mir-UL59	miR-UL59	guucucucgcucgucaugccgu	ULBP1 [35]	escape immune elimination
mir-UL69	miR-UL69	ccagaggcuaagccgaaaccg	N/A	N/A
mir-UL70	miR-UL70-3p	ggggaugggcuggcgcgcgg	MOAP1 [29]	inhibit mitochondria-induced apoptosis and the antiviral mechanism
			ERN1 [29]	
			PHAP1 [29]	
	miR-UL70-5p	ugcgucucggccucguccaga	N/A	N/A
mir-US4	miR-US4-3p	ugacagcccgcuacaccucu	ERAP 1[36]	N/A
			CASP7 [22]	
			CDK6 [22]	
	miR-US4-5p	uggacgugcagggggaugucug	PAK2 [37]	inhibit antigen presentation
			CASP2 [22]	
			ERAP1 [36]	
			QARS [36]	
mir-US5-1	miR-US5-1	ugacaagccugacgagagcgu	US7 [38]	escape the immune system; increase the production of infectious particles during the late phase of infection;
			VAMP3 [21]	
			RAB5C [21]	
			RAB11A [21]	
			SNAP23 [21]	
			CDC42 [21]	
			CDK6 [22]	
			FAS [22]	
			Gemini [39]	
			IKKα/β [23]	
mir-US5-2	miR-US5-2-3p	uaugauaggugugacgaugucu	US7 [38]	
			VAMP3 [21]	
			RAB5C [21]	
			RAB11A [21]	
			SNAP23 [21]	
			CDC42 [21]	
			CDK6 [22]	
			FAS [22]	
			NAB1 [31]	
	miR-US5-2-5p	cuuucgccacaccuauccugaaag	N/A	N/A
mir-US22	miR-US22-3p	ucgccggccgcgcuguaaccagg	US22 [40]	N/A
	miR-US22-5p	uguuucagcguguguccgcggg	US22 [40]	regulate apoptosis of host cells
			ATG5 [22]	
			EGR1 [41]	
mir-US25-1	miR-US25-1-3p	uccgaacgcuaggucgguucu	CDK6 [22]	reduce viral DNA synthesis
	miR-US25-1-5p	aaccgcucaguggcucggacc	Cyclin E2 [42]	
			BRCC 3[42]	
			EID1 [42]	
			MAPRE2 [42]	
			CD147 [42]	
			TRIM28 [42]	
mir-US25-2	miR-US25-2-3p	auccacuuggagagcucccgcggu	CASP3 [22]	
			CDK6 [22]	
			eIF4A1 [43]	
	miR-US25-2-5p	agcggucuguucagguggauga	N/A	
mir-US29	miR-US29-3p	cccacgguccgggcacaauca	N/A	N/A
	miR-US29-5p	uggaugugcucggaccgugacg	ATG5 [22]	regulate apoptosis of host cells
mir-US33	miR-US33-3p	ucacgguccgagcacauccaa	US29 [44]	N/A
	miR-US33-5p	gauugugcccggaccgugggcg	STX3 [45]	decreases the number of HCMV DNA copies
			CCND1 [22]	
15	26		N/A=No targets or exact function were found currently)	

List of pre-miRNAs and mature miRNAs. Previously reported 16 pre-miRNAs and 26 mature miRNAs encoded by HCMV were listed in this table, along with their potential targets and main functions

## Main text

### Methods of HCMV miRNA research and HCMV culture systems

HCMV can infect most organs and tissues in vivo, while its replication cycle differs significantly among different infected host cells. In fully-differentiated cells (such as fibroblasts, endothelial cells, and macrophages), HCMV undergoes lytic replication, while latent infection occurs in less-differentiated cells (CD14^+^ monocytes and CD34^+^ hematopoietic progenitor cells) [46, 47]. HCMV miRNAs may play an important role both in vivo and in vitro, as the expression kinetics of HCMV miRNAs are not exactly the same during different phases in the lytic or quiescent infection models [30, 48, 49]. However, given that virus-harboring monocytes are quite rare and there is no means of detecting or enriching HCMV-infected cells during natural latent infection so far, elucidating miRNA expression during latency faces multiple difficulties [50]. In recent years, primary myeloid cells have been receiving much attention due to the high ranges of latency-associated HCMV transcripts including miRNAs measured in this cell model, while much work on the potential targets of these HCMV miRNAs has been carried out [30, 31, 49]. Various experiments have been performed to elucidate the expression and functions of miRNAs during latency using primary myeloid cells infected with mutant viruses [21, 27, 31, 51, 52]. The mutation include altering the expression of a specific miRNA or removing the miRNA binding site. However, the main limitation of this system is the complexity of introducing mutations, as in the generation of mutants using the bacterial recombination method [52]. UV-inactivated virus is also widely used for comparison, in order to ensure that the miRNAs detected in latent models are derived from RNAs synthesized de novo during latent infection, as HCMV incorporates both viral and cellular RNA into virions [30, 49]. THP-1, a human monocyte cell line derived from a patient with acute monocytic leukemia, shares several characteristics with infected monocytes, so it is also widely used to investigate miRNA expression during latency [44, 53]. One of the major drawbacks of the THP-1 infection model is that representing a truly latent infection accurately may be difficult, because reactivation in these cells is ineffective. So the THP-1 cell line is not a substitute for monocytes to investigate HCMV miRNA expression during reactivation [54]. Besides, human embryonic lung fibroblasts, human foreskin fibroblasts, and human astrocytoma cells, among others, are used to model HCMV lytic replication [44, 55]. In fact, all currently known HCMV miRNAs have been detected in fibroblasts during lytic infection [40, 56]. To date, many studies have been published on the expression and kinetics of HCMV miRNAs in cell culture systems representing different HCMV life-cycles, but understanding the expression of HCMV miRNAs during the latent phase is needed to develop means of inhibiting reactivation of the virus from latency.

Besides HCMV infection systems, mouse cytomegalovirus (MCMV) and chimpanzee cytomegalovirus (CCMV) provide important model systems for HCMV pathogenesis as a result of the genetic similarity among MCMV, CCMV, and HCMV. In 2012, a group identified 17 rhesus CMV (RhCMV) miRNAs using next-generation sequencing [57]. One of these miRNAs, miR-Rh183–1, has been identified as a homolog of miR-US5–2-3p, which implies that RhCMV miRNAs may be investigated as supplements and references for the study of HCMV miRNAs. In contrast to RhCMV and CCMV, although HCMV and MCMV miRNAs may have the same targets, no sequence homology between HCMV and MCMV has been found so far [58].

### Mir-UL112

miR-UL112-3p is the most studied of all the HCMV miRNAs. In 2005, Pfeffer and colleagues used a new method of miRNA gene prediction combined with small-RNA cloning and first cloned mir-UL112 and another eight HCMV miRNA stem-loop sequences from several virus-infected cell types [9]. Pfeffer et al. also pointed out that miR-UL112-3p might target UL114, a homolog of the mammalian uracil-DNA glycosylase and concluded that miRNA expression might control the replication of viruses. miR-UL112-3p was later confirmed by independent groups [40, 55, 59]. Since then, the important role of miR-UL112-3p in processes such as escaping immune elimination and viral latency has been recognized. It binds to the 3′UTR of MICB, a stress-induced ligand recognized by the activating receptor NKG2D, at a site overlapping that of cellular microRNAs and inhibits MICB expression [17]. Thus, miR-UL112-3p may help HCMV to escape NKG2D-mediated immune detection and might be able to prevent the host from mutating that indispensable site [60, 61]. It has been reported that miR-UL112-3p subverts innate immunity by down-regulating type I interferon signaling, thus inhibiting natural killer cell cytotoxicity [62, 63]. Besides, miR-UL112-3p targets the pattern recognition receptor TLR2 (CD282), resulting in reduced stimulus-dependent ubiquitination of IRAK1 (an adaptor molecule downstream of TLR2 in the NF-κB pathway), which contributes to the establishment of latency [25]. And miR-UL112-3p down-regulates the level of BclAF1 (Bcl-2-associated transcription factor 1, a protein that restricts HCMV immediate early gene expression and spread) during the late phase of infection, thus, the absence of BclAF1 neutralization leads to inhibited viral gene expression and replication [15]. Moreover, miR-UL112-3p participates in the complex regulation of host cell metabolism [64]. miR-UL112-3p expression induces excessive proliferation of endothelial cells by up-regulating MAPK pathway-related genes or the cell growth-related genes TSPYL2, FXYD2, TAOK2, ST7L, and TP73 [20, 64], which might be one of the vital mechanisms of HCMV-induced endothelial dysfunction and may finally contribute to HCMV-mediated vascular diseases such as essential hypertension. Apart from targeting the transcripts of host cells, HCMV miR-UL112-3p also targets multiple viral transcripts such as IE72 (UL123, IE1), which plays an essential role in immune evasion to promote latent infection [18, 65]. The mechanism was confirmed in 2016 by removing the target site for miR-UL112-3p in the 3’UTR of IE72 [66]. Another functionally mature miRNA derived from the stem-loop sequence, miR-UL112-5p, was not detected until Stark and co-workers developed a new method for detecting HCMV miRNAs in 2012. They identified several previously unknown HCMV miRNAs including miR-UL112-5p using deep sequencing technology, which was groundbreaking and improved the accuracy of detecting true HCMV miRNAs [40]. miR-UL112-5p contributes to immune evasion by targeting endoplasmic reticulum aminopeptidase 1 (ERAP1), a key component of the antigen processing to decrease the CD8^+^ T cell response [26].

### Mir-UL148D

miR-UL148D is another HCMV miRNA that has been widely studied. It may play a role in the pathogenicity of clinical strains of HCMV by down-regulating the expression or inducing the degradation of several cellular genes [27, 28]. By targeting the UTR of RANTES (regulated upon activation normal T-cell expressed and secreted), miR-UL148D induces degradation of the human chemokine RANTES mRNA during infection to escape the immune response [27]. The chemokine RANTES is capable of attracting immune cells during inflammation and immune responses [67], and with the help of particular cytokines (i.e., IL-2 and IFN-γ) that are released by T cells, RANTES also induces the proliferation and activation of certain natural-killer cells to form CC-chemokine-activated killer cells [68]. And an miR-UL148D-specific inhibitor has been confirmed to inhibit the down-regulation of RANTES mediated by miR-UL148D, supporting the antisense agent as a therapeutic tool for HCMV infection [27]. Subsequently, researchers showed that HCMV also expresses miR-UL148D to induce apoptosis in host cells [28]. They found that HCMV-miR-UL148D binds with the 3′UTR of IEX-1 (immediately early gene X-1) to down-regulate its expression, leading to the apoptosis of human embryonic kidney 293 cells. In addition, researchers discovered that its apparent involvement in targeting the cellular receptor ACVR1B (activin receptor type-1B) in host cells helps HCMV to successfully establish latency [30].

### Mir-UL22A

In the year mir-UL22A was first cloned, the target genes of miR-UL22A-5p and miR-UL22A-3p were predicted; these included interferon 18 receptor precursor and histone 3 [56]. However, few further studies on these have been published. In 2015, a group found that BMPR2, a receptor involved in alternative polarization in macrophages, might be one of the target genes of miR-UL22A-5p [32]. Besides, according to this study, miR-UL22A-5p changes the levels of various proteins in MRC-5 fibroblasts transfected with this miRNA, such as HSPA8, a member of the HSP70 family of heat shock proteins, which is associated with the expansion of CMV-specific T cells and the immune clearance of viral replication in transplant recipients. A recent study showed that in infected CD34^+^ hematopoietic progenitor cells, miR-UL22A-5p and miR-UL22A-3p downregulate a mediator of TGF-β signaling, SMAD3, to maintain latency [31]. These findings imply that miR-UL22A plays a significant regulatory role in immune modulation.

### Mir-UL36

In 2005, Grey et al. first cloned the stem-loop sequence of mir-UL36 and four other sequences [59]. HCMV encodes but one intron miRNA (mir-UL36), which develops two functionally mature miRNAs (miR-UL36-3p and miR-UL36-5p) derived from the stem-loop sequence. Since then, several putative targets of HCMV-mir-UL36 have been discovered, of which HCMV UL138 is a new determinant of HCMV latent infection [33, 69]. Over-expression of miR-UL36-5p in HEK293 cells has been found to lead to an increase in HCMV DNA synthesis; it down-regulates HCMV UL138 protein expression and contributes to HCMV replication, indicating that miR-UL36-5p may be a viral miRNA contributing to HCMV replication [24]. Despite these findings, more physiological effects of miR-UL36-5p and miR-UL36-3p remain to be studied.

### Mir-UL59, mir-UL69 and mir-UL70

Meshesha and colleagues identified six mature miRNAs from four precursors, including mir-UL59–1 and mir-UL69, by deep sequencing and qPCR [56]. However, these two miRNAs were not functionally validated in this study, and no homologous sequence for mir-UL59 and mir-UL69 has been found in the CCMV genome. In addition, in Meshesha’s research, miR-UL70 was not detected by deep sequencing analyses. Similar to the cases of miR-UL59 and miR-UL69, since miR-UL70 was identified by northern blot analysis in 2005, it has not been functionally validated [40, 56, 59]. Therefore, the question has been raised whether they are true HCMV miRNAs. Furthermore, this implies that the accuracy of the deep sequencing technique may not be high as thought. Though the authenticity of these HCMV miRNAs remains to be established, commercially-designed probes for their amplification are available. Thus, caution should be exercised when discussing the research findings. In 2017, Ding et al. reported that they had identified miR-UL59 by RT-qPCR and that it may be closely associated with oral lichen planus, an autoimmune disease [35]. It may act by targeting the cellular gene ULBP1, which mediates the immune elimination of HCMV-infected cells, suggesting that miR-UL59 contributes to immune evasion. According to a recent report, HCMV expresses miR-UL70-3p and miR-UL148D to target the apoptotic genes MOAP1, ERN1, and PHAP1 and to inhibit mitochondria-induced apoptosis aa well as the antiviral mechanism [29]. Besides, miR-UL70-3p may regulate focal adhesions, gap junctions, MAPK signaling, and the Erb pathway, thus affecting epithelial cell migration and adhesion [70]. Another recent report has discovered 14 mature HCMV miRNAs in infected human lung fibroblasts, including miR-UL70-5p, indicating that HCMV virions and dense bodies carry miRNAs (such as miR-UL70-5p) which can be delivered to cells to affect cellular processes [71].

### Mir-US4

miR-US4-5p was found in 2005, and miR-US4-3p in 2012 [56, 59]. According to a report in 2011, besides miR-UL112-5p, miR-US4-5p is another miRNA that specifically down-regulates ERAP1 expression during infection, thus inhibiting the presentation of antigenic viral peptides to CD8^+^ T cells and contributing to HCMV survival at the immediate-early or early stage of infection [36]. In 2012, two independent studies conducted deep-sequencing analysis of HCMV miRNAs from infected human fibroblast cells, but the sequence of miR-US4-5p in these studies differed from that first identified in 2005 by 5 base pairs at the 5′ end [40, 56], which means that several papers were published using the wrong miRNA sequence and should be discussed with caution [37, 72, 73]. A recent study showed that miR-US4-5p targets PAK2 (p21-activated kinase 2) [37]. PAK2 is a protein that is widely expressed in all tissues and cell lines, and its signaling modulates apoptosis [74]. Thus, miR-US4-5p may participate in the modulation of apoptosis. Unfortunately, there has been little in-depth research on miR-US4-3p and miR-US4-5p, and previous work based on Grey’s results may need to be revisited.

### Mir-US5–1 and mir-US5–2

miR-US5–1 was discovered in 2005, together with miR-US5–2-3p, and miR-US5–2-5p was found in 2012 [9, 40]. However, their encoding transcripts have not been identified. miR-US5–1 and miR-US5–2-3p work synergistically. In 2011, it was reported that both miR-US5–1 and miR-US5–2-3p target US7, an endoplasmic reticulum-retained protein of unknown function, and act in a highly synergistic manner [38]. miR-US5–1 regulates US7 by binding to a completely complementary site within the 3′UTR of US7, resulting in strong repression of a reporter construct containing this 3′UTR. Intriguingly, they also found two binding sites for miR-US5–2-3p on the 3′UTR of US7. One site is completely complementary to the miR-US5–2-3p seed sequence, while the other is not perfectly matched. Binding of miR-US5–2-3p to the perfectly-matched site results in a decrease in transcript abundance, while binding to the other site does not. Their study was the first demonstration of a gene that is regulated via both mechanisms. The two miRNAs, miR-US-5-1 and miR-US5–2-3p, are derived from the same transcript due to their proximity and thus have synergistic action. Moreover, the two miRNAs also work together with other HCMV miRNAs. One study indicated that miR-US5–1, miR-US5–2-3p, and miR-UL112-3p coordinately target multiple members of the endocytic pathway, including VAMP3, RAB5C, RAB11A, SNAP23, and CDC42 [21]. Thus, these miRNAs and their corresponding target sequences cooperate to form a complex regulatory network to interfere with the release of pro-inflammatory cytokines (including interleukin-6 and tumor necrosis factor alpha) and help the virus to escape the immune system. During the late phase of HCMV infection, however, these miRNAs can also restructure components of the secretory pathway and form the virion assembly compartment where the final stages of HCMV particle formation occur to increase the production of infectious particles [21], suggesting that the function of miRNAs is dependent on the viral life-cycle. Other studies have also indicated that miR-US5–1 and miR-UL112-3p coordinately block proinflammatory cytokine production [23, 75]. In addition, miR-US5–1 has been shown to play a role in cell-cycle regulation; it directly targets the DNA replication inhibitor Geminin and its over-expression stimulates DNA synthesis in human brain glioma cells (U373), competitively inhibiting viral replication [39]. A recent study has shown that miR-US5–2 induces secretion of TGF-β and inhibits myelopoiesis in CD34^+^ HPCs by targeting the transcriptional repressor NAB1 [31]. These findings imply that miR-US5–1 affects viral replication and the host cellular environment by regulating the expression kinetics of host proteins during HCMV infection.

### Mir-US22

The two mature miRNAs derived from mir-US22 were discovered in 2012 by deep sequencing analysis [40]. It has been predicted that these miRNAs directly regulate the US22 transcript levels, and miR-US22-5p may target autophagy-related 5 (ATG5), a protein associated with autophagy and apoptosis, to modulate apoptosis [76]. Recently, EGR1 has been identified as a target of miR-US22 [41]. EGR1 regulates HCMV gene expression, and the down-regulation of the transcription factor mediated by miR-US22 may contribute to the reactivation of HCMV [41, 77].

### Mir-US25–1 and mir-US25–2

miR-US25–1-3p was identified 2 years after miR-US25–1-5p, and miR-US25–2-3p and miR-US25–2-5p were found in 2005 [9, 78]. It has been reported that US25–1-5p, US25–2-5p and US25–2-3p are highly expressed in THP-1 cells during HCMV latency, suggesting that they contribute to the establishment of latent infection [53]. However, Shen and colleagues reported that they only detected miR-US25–2-3p and miR-US5–1 in differentiated THP-1 cells [44]. Furthermore, in a later study, Lau et al. modeled infection with UV-inactivated virus and measured the HCMV miRNA levels [30]. They failed to identify miR-US25–1-3p and miR-US25–2-3p in CD14^+^ monocytes during latency but found them in lytically infected cells. Another interesting finding is that, in contrast to most HCMV miRNAs, many binding sites of miR-US25–1-5p are primarily within 5′UTRs [42]. This was the first demonstration that viral miRNA can target 5′UTRs. In addition, it has been shown that miR-US25–1-5p targets many genes involved in the cell cycle, tumor progression, and chromatin remodeling, such as cyclin E2, BRCC3, EID1, MAPRE2, and CD147, suggesting that it plays roles in the related pathways [42]. Chen and co-workers pointed out that miR-US25–1-5p may play an important role in chronic HCMV infection by targeting CD147 to antagonize the early innate immune response [52]. miR-US25–1-5p also contributes to aggravation of the apoptosis promoted by oxidized low-density lipoprotein in infected endothelial cells by targeting the 5′UTR of BRCC3 (BRCA1/BRCA2-containing complex subunit 3) and down-regulating its expression [79]. miR-US25–1-3p is down-regulated during chondrogenesis in human adipose-derived stem cells, indicating a complex regulatory circuit of HCMV miRNA during chondrogenesis of these cells [80]. In conclusion, these miRNAs may target genes that are important for the life-cycle of DNA viruses by reducing viral DNA synthesis, therefore driving the virus into latency and supporting immune evasion [14].

### mir-US29

MiR-US29-3p and miR-US29-5p were identified in 2012 [40]. MiR-US29-5p may be another HCMV miRNA that targets ATG5 to regulate apoptosis [22]. These two miRNAs may play important roles in the maintenance and reactivation of latency [48].

### Mir-US33

MiR-US33-5p was first described in 2005 while another mature miRNA derived from the same stem loop sequence was found 9 years later [44]. The US29 transcript is a potential target of miR-US33-3p, since pre-miR-US33 is encoded by sequences complementary to US29. miR-US33-5p directly targets the host gene Syntaxin 3 (STX3), which mediates zymogen granule-granule fusion and is positively associated with HCMV production [45]. The down-regulation of STX3 by miR-US33-5p significantly decreases the number of HCMV DNA copies, suggesting that miR-US33-5p expression facilitates the establishment or maintenance of HCMV latency.

## Conclusions

HCMV miRNAs have significant regulatory potential both alone and in combination, so it would be worthwhile to conduct an in-depth study of their targets and functions. Much work on their potential mechanisms has been carried out, while there are still some critical issues. Research methods for detecting unknown HCMV miRNAs and identifying their targets have improved over the past two decades. However, this field is hampered by many sub-par publications. Some miRNAs (miR-UL59, miR-UL69, and miR-UL70) have not yet been verified as true HCMV miRNAs, and several papers have been published using the wrong miR-US4 sequence. Besides, the significant RNA degradation that occurs during lytic infection may become a major obstacle to identifying their targets [9, 22], causing a bottleneck in further understanding the roles and functions of viral miRNAs. Further research is needed, and the conclusions of previous studies should be discussed with caution.

In summary, the goals are to study the miRNAs encoded by viruses and their roles in viral replication, expression, and infection, including but not limited to developing rapid and accurate detection methods for HCMV infection, to develop the planning of effective antiviral therapies, and to provide new molecular targets for the development of antiviral drugs. Recently, investigators have proposed a bioassay based on microgels with optical fluorescent oligonucleotide probes for the detection of circulating endogenous miR-US4-5p; this has improved the accuracy and reduced the cost of detecting HCMV infection [73]. The antagomir (also known as anti-miRs or blockmirs) miR-122 is now used to treat hepatitis C infection [81], and other antagomirs of virus-encoded miRNAs may be used to treat many viral diseases in the future.

## Data Availability

Not applicable.

## Abbreviations

ACVR1B: Activin receptor type-1B
ATG5: Autophagy-related 5
BclAF1: Bcl-2-associated transcription factor 1
BMPR2: Bone morphogenetic protein receptor type II
BRCC3: BRCA1/BRCA2-containing complex subunit 3
CASP2: Caspase-2
CASP3: Caspase-3
CASP7: Caspase-7
CCMV: Chimpanzee cytomegalovirus
CCND1: Cyclin D1
CDC42: Cell division control protein 42 homolog
CDK6: Cyclin-dependent kinase 6
dsDNA: Double-strand DNA
EGR1: Early growth response protein 1
EID1: EP300-interacting inhibitor of differentiation 1
eIF4A1: Eukaryotic initiation factor 4A-I
ERAP1: Endoplasmic reticulum aminopeptidase 1
ERN1: Endoplasmic reticulum to nucleus signaling 1
FXYD2: FXYD Domain Containing Ion Transport Regulator 2
HCMV: Human cytomegalovirus
HEK293: Human embryonic kidney 293 cells
HPCs: Hematopoietic progenitor cells
HSPA8: Heat shock 70 kDa protein 8
IE72: Immediate-early protein 72
IEX-1: Immediately early gene X-1
IFN-γ: Tumor necrosis factor γ
IKKα/β: IκB kinase α/β
IL-2: Interleukin 2
IL32: Interleukin 32
IRAK1: Interleukin-1 receptor-associated kinase 1
IRF1: Interferon regulatory factor 1
MAPK: Mitogen-activated protein kinase
MAP RE2: Microtubule-associated protein RP/EB family member 2
MCMV: Mouse cytomegalovirus
MICA: MHC class I polypeptide-related sequence A
MICB: MHC class I polypeptide-related sequence B
miRNA: microRNA
MOAP1: Modulator of apoptosis 1
MRC-5: Medical research council cell strain 5
NAB1: NGFI-A-binding protein 1
NF-κB: Nuclear factor kappa B
NKG2D: NK group 2 D
PAK2: p21-activated kinase 2
PHAP1: Putative HLA-DR-associated protein 1
QARS: Glutaminyl-tRNA synthetase
qPCR: Quantitative polymerase chain reaction
RAB11A: Ras-related protein Rab-11A
RAB5C: Ras-related protein Rab-5C
RANTES: Regulated on activation normal T cell expressed and secreted
RhCMV: Rhesus cytomegalovirus
RISC: RNA-induced silencing complex
RT-qPCR: Real-time polymerase chain reaction
SLC25A6(ANT3): Solute Carrier Family 25 Member 6(ADP/ATP translocase 3)
SNAP23: Synaptosomal-associated protein 23
ST7L: Suppression of tumorigenicity 7
STX3: Syntaxin 3
TAOK2: TAO kinase 2
TGF-β: Transforming growth factor β
TLR2: Toll-like receptor 2
TP73: Tumor protein 73
TRIM28: Tripartite motif-containing 28
TSPYL2: Testis-specific Y-encoded-like protein 2
ULBP1: UL16 binding protein 1
UTR: Untranslated region
VAMP3: Vesicle-associated membrane protein 3
